# Supplementary material for: Efficacy and cost effectiveness of intravenous ferric carboxymaltose versus iron sucrose in adult patients with iron deficiency anaemia
Source: PLoS One. 2021 Aug 10;16(8):e0255104. doi: 10.1371/journal.pone.0255104 (PMC8354439; doi:10.1371/journal.pone.0255104)
Supplement: Attachment — Submitted filename: Response to Reviewers.docx [file pone.0255104.s002.docx]

March 27th, 2021

The Editor-In-Chief

PLOS ONE

Dear Editor and Reviewers,

Revision of Manuscript: Manuscript ID PONE-D-20-35525

EFFICACY AND COST EFFECTIVENESS OF INTRAVENOUS FERRIC CARBOXYMALTOSE VERSUS IRON SUCROSE IN ADULT PATIENTS WITH IRON DEFICIENCY ANEMIA

The authors of the above manuscript would like to thank you for the email received on Jan 7^th^ 2021 which contained the valuable comments of anonymous reviewers and editorial office.

We are very pleased to know that it can be considered for publication contingent upon addressing the reviewers concerns and recommended edits. We really appreciate the time you spent reviewing this manuscript, and we thank you for all the comments. We have studied these comments carefully and have made corresponding corrections that we hope will meet with your approval. We believe once we addressed your comments, the quality of the manuscript will be improved.

Below kindly find our responses in regard to every comment mentioned by the reviewers.

At the end, we would like to thank the editor and the reviewers for considering this manuscript, and we look forward to hearing a favorable outcome from you soon. If you have any further queries, please do not hesitate to contact us.

Sincerely yours,

Dr Ahmed Basha

Corresponding author

Academic Editor’s and Reviewer(s)' Comments to Author:

From Academic Editor:

1. Rebuttal letter as a separate file labeled 'Response to Reviewers' (ok)
2. A marked-up copy of your manuscript that highlights changes made to the original version and should upload this as a separate file labeled 'Revised Manuscript with Track Changes'. (ok – file name: IDA_v10)
3. An unmarked version of your revised paper without tracked changes. You should upload this as a separate file labeled 'Manuscript'. (ok – file name: IDA_v12)
4. We suggest you thoroughly copyedit your manuscript for language usage, spelling, and grammar. The name of the colleague or the details of the professional service that edited your manuscript. A copy of your manuscript showing your changes by either highlighting them or using track changes (uploaded as a *supporting information* file) (ok- the ms has been sent for language editing by a professional company)
5. In your ethics statement in the manuscript and in the online submission form, please provide additional information about the patient records used in your retrospective study. Specifically, please ensure that you have discussed whether all data were fully anonymized before you accessed them. (have added)
6. Please ensure you have thoroughly discussed any potential limitations of this study within the Discussion section, including the potential impact of confounding factors.
7. In your Data Availability statement, you have not specified where the minimal data set underlying the results described in your manuscript can be found.
8. Upon re-submitting your revised manuscript, please upload your study’s minimal underlying data set as either Supporting Information files or to a stable, public repository and include the relevant URLs, DOIs, or accession numbers within your revised cover letter.
9. We note that you have not completed the financial disclosure section with a funding statement. Please include your amended statements within your cover letter.
10. Please complete your Competing Interests statement to state any Competing Interests.

Authors’ response:

1. The authors have prepared a rebuttal letter and labeled 'Response to Reviewers'
2. We have also submitted a separate file labeled 'Revised Manuscript with Track Changes'.
3. We submitted a cleaned version named as “Manuscript”
4. The authors have thoroughly reviewed the ms to edit the language, spelling and grammar.
5. It is confirmed that the patient records and all data used in our retrospective study were fully anonymized before we transferred to SPSS program for data analysis and reporting.
6. We have reviewed the discussion section and limitations of the study section to include any potential limitations including the effect of confounding factors.
7. Regarding the Data Availability statement, we have specified that the data is available upon request from the Senior/Corresponding Author. The minimal underlying data is submitted as Supporting Information files.
8. We have completed the financial disclosure statement with the funding statement.
9. We have also complete the Competing Interests statement.

General:

Comments to the Author

The manuscript must describe a technically sound piece of scientific research with data that supports the conclusions. Experiments must have been conducted rigorously, with appropriate controls, replication, and sample sizes. The conclusions must be drawn appropriately based on the data presented.

Authors’ response: We have carried out the study according to the CEA method using data retrospectively collected from the electronic patient medical record. It is not an experimental study. For further improvement, we have amended according to the reviewers’ suggestions/comments.

Has the statistical analysis been performed appropriately and rigorously?

Authors’ response: Appropriate stats methods have been done.

The data should be provided as part of the manuscript or its supporting information, or deposited to a public repository.

Authors’ response: Basic patient’s data is provided as requested. Detailed data in Excel sheet can be obtained from the primary upon request.

PLOS ONE does not copyedit accepted manuscripts, so the language in submitted articles must be clear, correct, and unambiguous. Any typographical or grammatical errors should be corrected at revision, so please note any specific errors here.

Authors’ response: The manuscript has been sent to professional editor (PED Ctr) for language editing service.

Reviewer: 1

Comments to the Author

The study is certainly important for health care providers in Qatar but I believe that some major limitations in the study design warrant a work-over of the study.

Authors’ response: we have included limitations of the study and suggested a few recommendations for future studies. As this study is a retrospective study design, future study using prospective data is recommended where more controls can be applied.

Please specify outcome measures in the abstract. Improvement in laboratory levels is a meaningless statement.

Authors’ response: We have elaborated and highlighted in red.

The complete patient population is poorly defined. IDA is a disease spectrum. I recommend to clarify the patient selection (hemoglobin, ferritin concentration and transferrin saturation).

Authors’ response: More information about the subjects were included to explain better the criteria/profiles of selection. We have included the criteria based on the Hb values.

Was the iron deficit calculated using the Ganzoni formula?

Authors’ response: Hemoglobin is done at hematology lab. Ferritin is an electrochemiluminescence immunoassay “ECLIA” done on the ROCHE cobas e 801 immunoassay analyzer based on sandwich principle. Iron saturation is a calculated parameter based on iron and transferrin. Iron Saturation% = Iron ÷(Transferrin x25.1)x100.

What kind of dosing strategy was used in the patient cohort?

Authors’ response: The therapeutic treatment dose for each patient depends on different criteria like weight and HB level. This was based on the international standard of regimen of FCM is between 25-100 mg IV daily PRN to a maximum cumulative dose of 1500 mg of iron, or 1-2 tablets of IS per day (to achieve 100-200 mg elemental iron daily in divided doses).

Was there a difference in total iron dose between groups?

Authors’ response: Yes, as the therapeutic treatment dose depends on different criteria like weight and HB level which is different from one patient to another. We have included the clarification under ‘Study design’.

Likewise, the exclusion criteria are poorly defended. How did the authors exclude other anemia causes?

Authors’ response: From the system (Cerner) as it's applicable to do filtration and sorting to the patient primary diagnose entered by the physician, based on this we include only IDA patient and exclude other types of anemia.

What kind of biochemical studies were routinely performed in the cohort?

Authors’ response: Maybe you mean biochemical parameters? FYI, all the parameters mentioned in the study were routinely monitored in the study cohort.

Please provide a dedicated table of the baseline characteristics to show any differences between groups at baseline.

Authors’ response: Table 1 is baseline demographic characteristics, which was analyzed. NS was found between the two treatment arms.

I have no experience in cost-effectiveness analyses and therefore cannot assess the statistical methods used.

Authors’ response: we have prepared according to the standard method of CEA and using appropriate statistical analysis. ICER a summary measure representing the economic value of an intervention, compared with an alternative (comparator) was further carried out. It reflects the incremental cost of the comparator when compared to current treatment, divided by the incremental benefit of the comparator compared to current treatment.

Reviewer: 2

Authors have here reported on the administration of two intra-venous iron formulations (ferric carboxy malthose and iron sucrose) in adult patients with iron deficiency anemia. This prospective study lasting 12 months is very interesting, well written and structured. Tables support the text. In detail this reviewer finds interesting the aspects related to RBC parameters improvement and cost (direct and indirect) analysis.

Authors’ response: Thank you. We will review and improve the quality of the article.

I suggest to modify some aspects of the paper :
I think the variation in Hgb and other RBCs parameters for both treatments should be better highlighted by a specific figure where I suggest to report in detail only haemoglobin levels, MCV and ferritin. I do not find useful reporting in table WBC and PLt counts, I do not see from data a particular high PLT count before treatment (reactive thrombocytosis) pre and post treatment values seem quite stable ,thus I think this should be just reported in the text.

Authors’ response: Thanks and I appreciate the suggestion. The authors feel that it will be easy for the readers to follow when presented in a table. We have mentioned the significant of Hgb, MCV and ferritin in the discussion section.

Further, it seems that patients treated with iron sucrose had baseline Hgb levels lower than patients treated with ferric carboxymalthose. Could please authors better describe this aspect?

Authors’ response: Yes, it is true and in the calculation we have applied the difference in relation to the baseline values. Thus, the increment was used in the calculation.

Authors should also better afford the issues related to oral iron in comparison to iv iron.

Authors’ response: Yes, we acknowledged the cost of oral iron in comparison to IV ron formulation. However, in the long run and taking into consideration of all aspects in patient management, at least in this study, the higher cost of FCM versus IS can be offset by savings in healthcare personnel time and bed space. The overall cost was slightly higher in IS group. The treatment with FCM and IS resulted a mixed finding.

References should be updated with a recently published article referring to the administration of iv iron vs oral iron (10.1007/s00277-020-04361-3)

Authors’ response: we have added an additional ref regarding to the administration of IV iron vs oral iron. i.e Giordano, G., Napolitano, M., Di Battista, V. et al. Oral high-dose sucrosomial iron vs intravenous iron in sideropenic anemia patients intolerant/refractory to iron sulfate: a multicentric randomized study. Ann Hematol (2020). https://doi.org/10.1007/s00277-020-04361-3

Reviewer: 3

In this study, the authors evaluate the efficacy and cost-effectiveness of ferric carboxymaltose compared to iron sucrose in adult patients with iron deficiency anemia.

Although clinically relevant due to the frequency of the disease and its impact, the work presents several major limitations:
- consider to reorganize the structure of the introduction (role of oral iron and intravenous iron) and include more References since many data are not supported by references (e.g., “Intravenous iron preparations have been used for treating IDA, with a promising result and making it possible to avoid blood transfusion and side effects of the oral iron preparation.” “Iron sucrose (IS) has been widely used for treating IDA that shows the potential result.”)

Authors’ response: We have included in the discussion section which is more interesting to include when the findings are known. We have added another 2 references/studies (25, 26) in relation to these points.

- The primary outcome, i.e., the treatment efficacy, is not well defined. Indeed, the authors consider hematological and iron parameters at a follow-up time point that was not clearly defined.

“The post level was measured at least 2 weeks after the medication administration.” The authors do not specify the follow-up length (median or mean days of follow-up). This is relevant since the hemoglobin levels and iron parameters 2 weeks after the administration cannot be compared to values at a longer follow-up (e.g., 40 days). At least the follow-up between the two groups should be similar. Moreover, iron parameters at 2 weeks are still disrupted by the IV iron administration.

Authors’ response: we have included the above concern as a limitation and recommended for careful observation in a prospective study. Thanks for raising this matter.

- The total amount of administered iron per patient in the two groups should be specified in order to compare the efficacy parameters.

Authors’ response: It's already specified in the data collection sheet. The total dose for each patient is calculated in both arms.

- The two groups (FCM vs. IS) characteristics are not well defined: the total amount of iron administered per patient, causes of iron deficiency anemia, clinical symptoms. Moreover, their baseline hemoglobin levels are different. I suggest commenting on this difference and its potential impact on therapy choice. Nevertheless, since hemoglobin is the efficacy parameter, and hemoglobin increase is used for the cost-effectiveness evaluation, the authors should consider potential adjustments and comment on this.

Authors’ response: Yes, as the therapeutic treatment dose depends on different criteria like weight and HB level which is different from one patient to another

- Adverse events data are not presented (even if not adverse events have been observed should be specified), while in the Methods section, there is a specific paragraph about the management of adverse reaction. Moreover, no data about hypophosphatemia have been presented. I would consider editing the part about drugs used to manage these reactions since the role of antihistaminic drugs is controversial.

Authors’ response: Hypophosphatemia is the most common adverse reaction happened with IV iron supplement but not serious like anaphylactic reactions so we didn't mention it and focus only on hypersensitivity as we are doing in our practice.

(The following adverse drug reactions and incidences are derived from product labeling unless otherwise specified.

>10%: Endocrine & metabolic: Hypophosphatemia (<2 mg/dL: 27%; transient)

1% to 10%:

Cardiovascular: Increased systolic blood pressure (6%; transient), flushing (4%), hypertension (4%), hypotension (1%)

Dermatologic: Skin discoloration at injection site (1%)

Gastrointestinal: Nausea (7%), vomiting (2%), dysgeusia (1%)

Hepatic: Increased serum alanine aminotransferase (1%)

Nervous system: Dizziness (2%), headache (1%)

.

Minor revisions:
- Introduction, page 4: the sentence “Anemia is the most common nutritional deficiency” should be revised. Indeed, iron deficiency is the most common nutritional deficiency.

Authors’ response: We have edited the text.

- Introduction, page 4: “poor appetite” is not considered one of the most frequent symptoms of iron deficiency anemia

Authors’ response: We have taken this symptom out from the text.

- Check all the abbreviations: some are spelled out many times, some are not spelled out

Authors’ response: we have maintained the consistency; abbreviations were spelled out once, and then abbreviated throughout the text.

- “The acquisition cost of FCM is expensive but reported to have a better outcome.”: compared to?

Authors’ response: We have added IS and other agents in the text (highlighted) for clarification. Further, in the analysis, ICER was carried out in such situation where a treatment is more expensive but have better outcome.

- Units of measurements are not always specified in the tables

Authors’ response: Thanks. We have added in several places in the tables.

- Native English revision is required

Authors’ response: thanks and we have sent to a professional company (Proofreading and Editing Service, PES Center) for language editing service.
