# Supplementary material for: Efficacy and cost effectiveness of intravenous ferric carboxymaltose versus iron sucrose in adult patients with iron deficiency anaemia
Source: PLoS One. 2021 Aug 10;16(8):e0255104. doi: 10.1371/journal.pone.0255104 (PMC8354439; doi:10.1371/journal.pone.0255104)
Supplement: Attachment — Submitted filename: Response to reviewers_June .docx [file pone.0255104.s003.docx]

June 30th, 2021

The Editor-In-Chief

PLOS ONE

Dear Editor and Reviewers,

Revision of Manuscript: Manuscript ID PONE-D-20-35525

EFFICACY AND COST EFFECTIVENESS OF INTRAVENOUS FERRIC CARBOXYMALTOSE VERSUS IRON SUCROSE IN ADULT PATIENTS WITH IRON DEFICIENCY ANEMIA

The authors of the above manuscript would like to thank you for the email received which contained a few more comments for improvement.

We are very pleased to know that it can be considered for publication contingent upon addressing the reviewers concerns and recommended edits. We really appreciate the time you spent reviewing this manuscript, and we thank you for all the comments. We have studied these comments carefully and have made corresponding corrections that we hope will meet with your approval. We believe once we addressed your comments, the quality of the manuscript will be improved.

Below kindly find our responses in regard to every comment mentioned by the reviewers.

At the end, we would like to thank the editor and the reviewers for considering this manuscript, and we look forward to hearing a favorable outcome from you soon. If you have any further queries, please do not hesitate to contact us.

Sincerely yours,

Dr Ahmed Basha

Corresponding author

Reviewers' comments:

Reviewer's Responses to Questions

**Comments to the Author**

1. If the authors have adequately addressed your comments raised in a previous round of review and you feel that this manuscript is now acceptable for publication, you may indicate that here to bypass the “Comments to the Author” section, enter your conflict of interest statement in the “Confidential to Editor” section, and submit your "Accept" recommendation.

Reviewer #2: All comments have been addressed

Reviewer #3: (No Response)

2. Is the manuscript technically sounds, and do the data support the conclusions?

The manuscript must describe a technically sound piece of scientific research with data that supports the conclusions. Experiments must have been conducted rigorously, with appropriate controls, replication, and sample sizes. The conclusions must be drawn appropriately based on the data presented.

Reviewer #2: Yes

Reviewer #3: Partly

3. Has the statistical analysis been performed appropriately and rigorously?

Reviewer #2: Yes

Reviewer #3: I Don't Know

4**. Have the authors made all data underlying the findings in their manuscript fully available?**

The [PLOS Data policy](http://www.plosone.org/static/policies.action#sharing) requires authors to make all data underlying the findings described in their manuscript fully available without restriction, with rare exception (please refer to the Data Availability Statement in the manuscript PDF file). The data should be provided as part of the manuscript or its supporting information, or deposited to a public repository. For example, in addition to summary statistics, the data points behind means, medians and variance measures should be available. If there are restrictions on publicly sharing data—e.g. participant privacy or use of data from a third party—those must be specified.

Reviewer #2: Yes

**Reviewer #3: No**

**Authors’ response: Dear Editor and Reviewer, The authors have submitted an excel sheet which contain all the data in our previous submission. Kindly, check and let us know if the sheet cannot be opened or found.**

5. Is the manuscript presented in an intelligible fashion and written in standard English?

PLOS ONE does not copyedit accepted manuscripts, so the language in submitted articles must be clear, correct, and unambiguous. Any typographical or grammatical errors should be corrected at revision, so please note any specific errors here.

Reviewer #2: Yes

Reviewer #3: Yes

6. Review Comments to the Author

Please use the space provided to explain your answers to the questions above. You may also include additional comments for the author, including concerns about dual publication, research ethics, or publication ethics. (Please upload your review as an attachment if it exceeds 20,000 characters)

Reviewer #2: I have revised this version of the manuscript, I think it has been improved , all comments have been well addressed. I think the paper can now be accepted for publication

**Reviewer #3: Considering the authors' comments at first revision, some crucial points have not been addressed:
- The author did not specify the follow-up length and this dramatically affect the comparison of efficacy parameters
- The authors did not specify the mean/median amount of iron (in milligrams) administered in the two groups and this dramatically affect the efficacy outcomes.
- The different Hb baseline level could affect therapeutic decisions
- The conclusion that IS showed a higher increase in Hb is affected by the Hb baseline difference between the two groups**

**- The author did not specify the follow-up length and this dramatically affect the comparison of efficacy parameters**

**Authors’ response: Dear reviewer, as stated in the method section, the authors have explained that the follow-up length was between June 2017 till Apr 2019 (around 23 months):**

“A retrospective study was conducted to review the efficacy and cost effectiveness of intravenous (IV) ferric carboxymaltose (FCM) versus iron sucrose (IS) in adult patients with iron deficiency anemia (IDA) who are following with medical city IV suite between 01/01/2018 – 30/11/2018. They were included in the study and their labs including; **hemoglobin, ferritin and TSAT were followed within the period** from 01/06/2017 to 01/04/2019”

The study compared the response to the therapy using the improvement in **hemoglobin, ferritin and TSAT** levels to evaluate the effectiveness of FCM and IS in treating IDA patients, **hemoglobin, ferritin and TSAT** and other parameters that were obtained 6 months before and 4 months after the two therapies within the period from 01/06/2017 to 01/04/2019.

**Action: we will add ’23 months’ in brackets and the follow-up period in the data collection section.**

**- The authors did not specify the mean/median amount of iron (in milligrams) administered in the two groups and this dramatically affect the efficacy outcomes.**

**Authors’ response: The information was in the original ms submitted earlier. We have now highlighted for clarification. The information can be found in the study design section.**

**- The different Hb baseline level could affect therapeutic decisions**

**Authors’ response: Further, regarding the statistical analysis, we have ran analysis to control for the data at baseline and control for any differences. In the statistical analysis section, we wrote:**

**Efficacy measures included change in Hgb, ferritin, and TSAT from the baseline and were calculated using Wilcoxon signed rank test. Two-Way Repeated Measures ANOVA (Mixed-Factor ANOVA) was applied to compare between before and after effect in the two treatment arms (Table 3).**

**Further, we looked and measured the changes from the baseline. Thus, this will overcome the different baseline level of lab data (Table 3).**

**- The conclusion that IS showed a higher increase in Hb is affected by the Hb baseline difference between the two groups**

**Authors’ response: Our conclusion is justified based on the statistical analysis and considerations taken in the analysis - % change/improvement and ANOVA method used. We hope these explanations have satisfied your concerns above.**

7. PLOS authors have the option to publish the peer review history of their article ([what does this mean?](https://journals.plos.org/plosone/s/editorial-and-peer-review-process#loc-peer-review-history)). If published, this will include your full peer review and any attached files.


If you choose “no”, your identity will remain anonymous but your review may still be made public.


**Do you want your identity to be public for this peer review?** For information about this choice, including consent withdrawal, please see our [Privacy Policy](https://www.plos.org/privacy-policy).

Reviewer #2: No

Reviewer #3: No

[NOTE: If reviewer comments were submitted as an attachment file, they will be attached to this email and accessible via the submission site. Please log into your account, locate the manuscript record, and check for the action link "View Attachments". If this link does not appear, there are no attachment files.]
